# Cholesterol Forms and Traditional Lipid Profile for Projection of Atherogenic Dyslipidemia: Lipoprotein Subfractions and Erythrocyte Membrane Cholesterol

**DOI:** 10.1007/s00232-013-9611-2

**Published:** 2013-11-16

**Authors:** Hüseyin Avni Uydu, Mehmet Bostan, Mehtap Atak, Adnan Yılmaz, Adem Demir, Buket Akçan, Fatih Sümer, Nimet Baltaş, Zakir Karadağ, Yavuz Uğurlu, Asım Örem

**Affiliations:** 1 Department of Medical Biochemistry, Faculty of Medicine, University of Recep Tayyip Erdoğan, Rize, Turkey; 2 Department of Cardiology, Faculty of Medicine, University of Recep Tayyip Erdoğan, Rize, Turkey; 3 Department of Chemistry, Faculty of Arts and Sciences, University of Recep Tayyip Erdoğan, Rize, Turkey; 4 Department of Medical Biochemistry, Faculty of Medicine, Karadeniz Technical University, Trabzon, Turkey; 5 Department of Internal Medicine, Faculty of Medicine, University of Recep Tayyip Erdoğan, Rize, Turkey

**Keywords:** Coronary artery disease, Dyslipidemia, Erythrocyte cholesterol, Lipoprotein subfractions

## Abstract

Atherogenic dyslipidemia characterized by abnormal changes in plasma lipid profile such as low high-density lipoprotein (HDL) and increased triglyceride (TG) levels is strongly associated with atherosclerotic diseases. We aimed to evaluate the levels of pro- and antiatherogenic lipids and erythrocyte membrane cholesterol (EMC) content in normo- and dyslipidemic subjects to investigate whether EMC content could be a useful marker for clinical presentation of atherogenic dyslipidemia. Low-density lipoprotein (LDL), HDL and their subfraction levels and erythrocyte lipid content were determined in 64 normolipidemic (NLs), 42 hypercholesterolemic (HCs) and 42 mixed-type dyslipidemic subjects (MTDs). Plasma atherogenic lipid indices [small–dense LDL (sdLDL)/less-dense HDL (LHDL), TC/HDL-C, TG/HDL-C and Apo B/AI] were higher in MTDs compared to NLs (*p* < 0.001). The highest sdLDL level was observed in HCs (*p* < 0.01). Despite a slight increase in EMC level in dyslipidemic subgroups, the difference was not statistically significant. A significant negative correlation, however, was observed between EMC and sdLDL/LHDL in HCs (*p* < 0.035, *r* = −0.386). Receiver operating characteristic curves to predict sdLDL level showed that TG and EMC levels had higher area under curve values compared to other parameters in HCs. We showed that diameters of larger LDL and HDL particles tend to shift toward smaller values in MTDs. Our results suggest that EMC content and TG levels may be a useful predictor for sdLDL level in hypercholesterolemic patients.

## Introduction

Atherogenic dyslipidemia is one of the main contributing factors for the progress of cardiovascular complications and is characterized with increased plasma triglyceride (TG) and low-density lipoprotein (LDL-C) levels and decreased high-density lipoprotein (HDL-C) levels (Krauss 2005; Musunuru 2010). Hypercholesterolemia and mixed-type dyslipidemia are the most common forms of atherogenic dyslipidemia; in hypercholesterolemia the plasma level of total cholesterol (TC) is high while in mixed-type dyslipidemia both TC and TG levels are elevated (Jia et al. 2007). It has been demonstrated by numerous studies over the last two decades that plasma lipid profiling using routine methods fails to distinguish lipid and lipoprotein abnormalities associated with cardiovascular diseases (CAD). It has been increasingly evident that analysis of lipoprotein subfractions rather than total plasma lipoprotein measurement is more informative in risk assessment for cardiovascular complications. LDL and HDL lipoproteins exhibit a heterogenic distribution ranging from small, dense to larger and lighter particular structure in standard fractionation methods (Superko 2009). Pro- and antiatherogenic properties of LDL and HDL depend on their particle size and on plasma concentration (Maeda et al. 2012; Musunuru 2010; Superko 2009). Recent experimental and epidemiological reports showed that small-sized LDL and HDL particles [Small-Dense LDL (sdLDL) and HDL_3c_ (SHDL)] are crucial players in pathophysiology of atherogenesis compared to larger particles [Large Buoyant LDL (LbLDL) and HDL_2b_ (LHDL)]. Particularly, sdLDL, which are more susceptible to oxidation and conformational changes, accelerates inflammatory reactions and are associated with atheromatous plaque formation (Gou et al. 2005; Jia et al. 2007; Kwon et al. 2006; Mohan et al. 2005; Musunuru 2010; Shoji et al. 2009; Superko 2009).

The lipid structure of erythrocyte membrane differs greatly from those of other cells because erythrocytes lack lipoprotein receptors and *de novo* cholesterol synthesis (Tziakas et al. 2010). Although erythrocyte membrane cholesterol (EMC) content is correlated with plasma lipid composition, the mechanism concerning a possible interaction of EMC with the lipoprotein subfractions is not yet clear. Recent reports indicate that EMC constitutes a substantial bulk of the cholesterol in lipid-rich core regions of atherosclerotic plaques and it might serve as a valuable marker for cardiovascular events (Tziakas et al. 2008, 2010, 2012; Vaya et al. 2008).

The aim of the present study was to elucidate the tendency of individuals with lipidemia to develop atherogenic dyslipidemia by analyzing plasma lipid and lipoprotein subfractions and to examine whether EMC has predictive potency for plasma levels and composition of pro- and antiatherogenic lipoproteins. To our knowledge, this is the first study investigating the association of EMC content with lipoprotein subfractions.

## Materials and Methods

### Study Design

A total of 165 participants, 86 men and 79 women, were volunteered and enrolled onto the study. Participants were recruited by Cardiology Department of the Medical School at Recep Tayyip Erdoğan University in Turkey. All participants went through a screening protocol which included evaluation of their medical history and testing for full hematologic and biochemical profiles. Individuals with hypothyroidism, renal insufficiency, hepatic dysfunction, malignant diseases or ongoing infections were excluded from the study. Seventeen patients were excluded because of aforementioned illnesses, abnormal erythrocyte counts and missed blood samples. Patients were eligible to take part in the study if they met the criteria of the National Cholesterol Education Program-Adult Treatment Panel 3 (NCEP-ATP 3) and were not receiving lipid-lowering therapy in the previous 1 month before the entry into the study. The research was approved by the local ethics committee, and all participants gave written consent before entering the study. Qualified participants were classified into three groups according to their plasma TC and TG levels: 64 normolipidemic subjects with TC level of <240 mg/dL and TC level <200 mg/dL (NLs); 42 hypercholesterolemic subjects with TC levels above 240 mg/dL (HCs); and 42 mixed-type hyperlipidemic subjects with TC levels of ≥240 mg/dL and TG levels of ≥200 mg/dL [mixed-type dyslipidemic subjects (MTDs)]. Diabetes was defined as fasting serum glucose values of TC ≥ 126 mg/dL on 2 or more occasions, or 2-h serum glucose level of ≥200 mg/dL after an oral glucose tolerance test. The diagnosis of hypertension was based on a known history and systolic blood pressure of ≥ 140 mm Hg or diastolic blood pressure of ≥ 90 mm Hg. All measurements were done at least three times.

### Measurement of Serum Lipid and Lipoprotein Levels

Blood samples were drawn after a 12 h fasting period and plasma was separated by low-speed (2500×*g*) centrifugation for 15 min. Plasma levels of TC and TG were determined with enzymatic methods using a Abbot Architect C16000 autoanalyzer with supplied reagents (Diamond Diagnostics Inc., Boston, USA). Total HDL-C was measured by the dextran sulphate-Mg^+2^ precipitation method, and the total LDL-C was calculated using the Friedewald’s formula as all participants had plasma TG levels in the range of 41–365 mg/dL. Apolipoprotein A-I (Apo A-I), B (Apo B), E (Apo E) and lipoprotein (a) [Lp (a)] levels were assessed by immunonephelometry using Date Behring kit (BN II, Marburg, Germany).

### Measurement of LDL and HDL Subfractions

LDL and HDL subfraction analysis were carried out electrophoretically by using high-resolution 3 % polyacrylamide gel tubes and the Lipoprint System (Quantimetrix) according to manufacturer’s instructions (Kim et al. 2008). Lipoprint System separated lipoproteins on the basis of net surface charge and size of LDL and HDL. LDL particles were categorized into LbLDL and sdLDL species as previously described (Vega et al. 2003). The mean LDL particle size greater than 263 `Å was defined as pattern A phenotype and those with smaller size as pattern B phenotype. Five subclasses based on HDL particle size has been described previously (Muth et al. 2010; Superko 2009). HDL phenotypes were classified as small HDL (SHDL), intermediate HDL (IMHDL) and less HDL (LHDL) (Lagos et al. 2009). All subfractions were expressed as mg/dL.

### Measurement of Erythrocyte Membrane Lipids

Erythrocytes were separated from plasma and the buffy coat containing leucocytes and platelets by sedimentation (3,000×*g,* 10 min) at 4 °C and washed three times with phosphate-buffered saline (PBS). Erythrocyte ghost membrane was prepared as described previously (Dodge et al. 1963). Extraction of membrane lipid was done using the procedure developed by Rose and Oklander (1965), in which lipids are extracted from an aliquot of erythrocyte membrane suspension with chloroform-isopropanol (7:11, v/v). Membrane cholesterol was assayed by cholesterol oxidase method using a high-performance Monotest kit (Boehringer Mannheim, Mannheim, FRG) (Ott et al. 1982). Membrane total phospholipids was estimated as described by Bartlett (Bartlett 1959). Bradford protein assay was used to measure protein concentration of membrane (Bradford 1976).

### Statistical Analysis

Data are presented as mean ± SD for normally distributed continuous variables, as median (inter-quartile range, IQR) for nonnormally distributed continuous variables and as percentages for categorical variables. Normality was tested using the Kolmogorov–Smirnov test. Differences between the values among lipidemic subgroups were evaluated by one-way analysis of variance with least significant difference or Kruskal–Wallis with Mann–Whitney post hoc tests as appropriate. Analysis of variance with covariates (ANCOVA) was used to evaluate differences in lipids, LDL subfractions and EMC levels between the study groups after adjustment for all the variables that were significantly different among the subgroups. Comparisons between categorical variables were performed with Chi square test or Fisher’s exact test as appropriate. Spearman’s correlation analysis was performed to estimate the relationship between atherogenic lipid indexes. Seventy-fifth percentiles of the overall sdLDL, pattern B, EMC, TC/HDL-C and TG/HDL-C ratios in all groups were taken as a cut-off for defining elevated levels of these variables. Receiver operating characteristic (ROC) curve was constructed for diagnosis of sdLDL with various lipid parameters. Cut-off values were selected on the basis of optimum sensitivity and specificity. The area under the ROC curve (AUC) is considered to be a useful quantitative measure of accuracy. Group differences with *p* values of < 0.05 were accepted significant. The SPSS 16.0 statistical software package (SPSS Inc., Chicago, IL, USA) was used for all calculations.

## Results

**Fig. 1:**
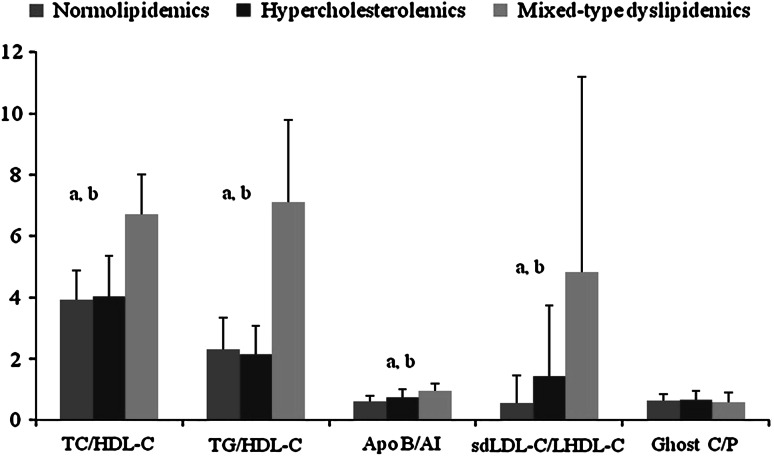
Presentation of lipid, lipoprotein and apoprotein ratios as a dyslipidemia indicator in all groups. Comparison of normolipidemic group with mixed-type dyslipidemic (**a**) and hypercholesterolemic group with mixed-type dyslipidemic (**b**) group; *p* < 0.001

Demographic characteristics were similar in all study groups, except for smoking and gender as there were more male participants in MTDs (*p* < 0.05) (Table 1). Table 2 gives the unadjusted results of comparisons among the subgroups regarding plasma lipid profile, lipoprotein subfractions and erythrocyte cholesterol. Consistent with how participants were categorized in this study, i.e., according to plasma TC and TG levels, higher plasma TC and TG levels were found in MTDs while HCs had higher TC levels compared to NLs (*p* < 0.01). The same trend was observed for other lipidic parameters. Decreased LDL peak size (<263 Å) and increased sdLDL level (42 mg/dL) were more apparent in MTDs compared to other groups. Plasma Lp (a) and erythrocyte cholesterol levels were slightly higher in MTDs, but the difference was not statically significant. HDL subfractionation showed that SHDL was high only in HCs (8 mg/dL) whereas LHDL was lower in MTDs (10 mg/dL) but not significantly (Table 2). ANCOVA analyses demonstrated that sdLDL level and mean LDL size remained significantly higher in MTDs after adjustment for gender and smoking that were significantly different between subgroups. In view of our results reported herein, the proportions of pro- and antiatherogenic plasma lipids were considered as dyslipidemic indicators (Fig. 1). All ratios except for erythrocyte C/P were significantly higher in MTDs compared to other groups (*p* < 0.01). In addition, the segregation test indicated certain dyslipidemic indices that were strongly associated with lipid atherogenity (Table 3). Higher incidence of TC/HDL-C ratio was more pronounced in dyslipidemic subgroups (*p* < 0.001) and it was almost 98 % in MTDs. High frequencies of plasma sdLDL level and pattern B phenotype were clearly associated with dyslipidemic subgroups. Moreover, an intriguing finding in our study was that both dyslipidemic subgroups, especially MTDs, had a higher incidence of high EMC content.

**Table 1:** Distribution of baseline characteristics of normolipidemic and dyslipidemic groups

Variable	Normolipidemic group (*n* = 64)	Hypercholesterolemic group (*n* = 42)	Mixed-type dyslipidemic group (*n* = 42)
Age (years), mean ± SD	58 ± 11	58 ± 13	56 ± 11
Body mass index (kg/m^2^), mean ± SD	29 ± 5	29 ± 5	30 ± 5
Waist circumference (cm), mean ± SD	101 ± 13	100 ± 11	106 ± 11
Gender, male (%) (*n*)	64 (41)	33 (14)	67 (28)^b^*
Hypertension (%) (*n*)	42 (27)	43 (18)	43 (18)
Smoking, % (*n*)	16 (10)	16 (7)	42 (18)^a^*^,b^*
Diabetes (%) (*n*)	16 (10)	11 (5)	23 (12)

Comparison of mixed-type dyslipidemic group with normolipidemic (a) and hypercholesterolemic groups (b)

*p < 0.05

**Table 2:** Concentrations of plasma and erythrocyte lipids and plasma apolipoproteins

Variable	Normolipidemic group (*n* = 64)	Hypercholesterolemic group (*n* = 42)	Mixed-type dyslipidemic group (*n* = 42)
TC (mg/dL)	164 ± 24	249 ± 25^a^***	242 ± 28^b^***
TG (mg/dL)	94 (84–99)	108 (99–120)	239 (228–272)^b^***^,c^***
LDL-C (mg/dL)	104 ± 24	158 ± 27^a^***	157 ± 30^b^***
HDL-C (mg/dL)	44 (40–48)	50 (47–60)^a^**	37 (35–41)^b^**^,c^***
VLDL-C (mg/dL)	41(35–42)	52 (49–61)^a^***	59 (52–65)^b^***
Apo B (mg/dL)	91 ± 20	121 ± 26^a^***	130 ± 29^b^***
Apo AI (mg/dL)	148 ± 27	157 ± 28^a^***	135 ± 2^c^***
Lp (a) (g/L)	16 (15–35)	25 (24–62)	20 (8–42)
Apo E (mg/dL)	4.1 (3.8–4.6)	4.0 (3.7–4.6)	5.8 (5.1–6.0)^b^***^,c^***
Buoyant LDL-C (mg/dL)	50 (36–49)	39 (37–55)	37 (34–43)
sdLDL-C (mg/dL)	4 (5–10)	7 (10–26)	42 (37–56)^b^***^,c^**
Mean LDL size (Å)	268 (267–269)	268 (264–268)	263 (260–272)^b^***^,c^*
LHDL-C (mg/dL)	13 (15–18)	17 (16–22)	10 (9–13)^b^***^,c^***
SHDL-C (mg/dL)	5 (5–7)	8 (6–9)^a^**	6 (4–7)^c^*
Erythrocyte cholesterol (μg/mg prt.)	436 ± 135	461 ± 205	490 ± 221
Inorganic phosphate (μg/mg prt.)	703 ± 32	716 ± 34	712 ± 31

Data are presented as mean ± SD for normally distributed and as median (IQR) for nonnormally distributed continuous variables

Comparison of normolipidemics with hypercholesterolemics (a), mixed-type dyslipidemics (b), and hypercholesterolemics with mixed-type dyslipidemics (c)

*p < 0.0 5, **p < 0.01, ***p < 0.005

**Table 3:** Prevalence of different forms of atherogenic dyslipidemia in study groups

Sample group	High TC/HDL-C ratio, % (*n*)	High TG/HDL-C ratio, % (*n*)	High sdLDL % (*n*)	Pattern B % (*n*)	High erythrocyte C/P ratio % (*n*)
Normolipidemic group (*n* = 64)	20 (13)^a^***	16 (10)^a^	6 (4)^a^	0 (0)^a^	0 (0)^a^
Hypercholesterolemic group (*n* = 42)	64 (27)^b^***	12 (5)^a^	26 (11)^b^*	7 (3)^b^*	15 (6)^b^**
Mixed-type dyslipidemic group (*n* = 42)	98 (41)^c^***	100 (42)^b^***	50 (21)^b^***	17 (7)^b^**	15 (6)^b^**

The 75th percentiles of sdLDL, pattern B, EMC, and proportions of TC/HDL-C and TG/HDL-C were assumed as a cutoff for defining elevated levels of these variables

^a,b,c^Significant difference within a column at *p < 0.0 5, **p < 0.01, and ***p < 0.001

**Fig. 2:**
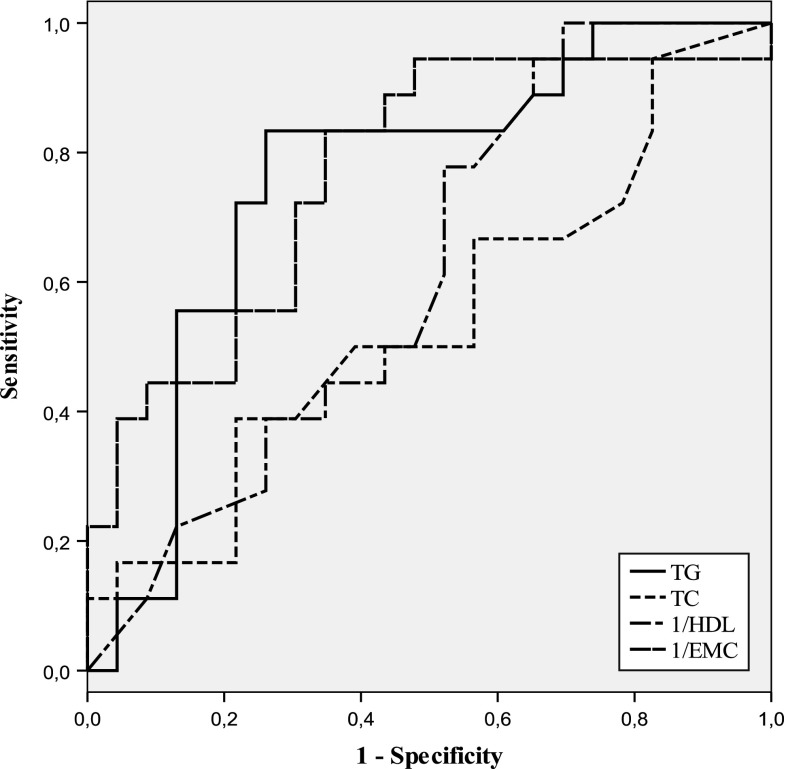
Receiver-operating characteristics (ROCs) curve analysis regarding predictive accuracy to determine the presence of high sdLDL level in subjects with hypercholesterolemia

To elucidate a possible relationship between lipoproteins and EMC content, Spearman correlation analysis was applied to the data and revealed a negative correlation between erythrocyte C/P ratio and sdLDL/LHDL ratio (*r* =−0.386, *p* < 0.05) only in HCs (Table 4). In addition, a strong positive correlation was found between sdLDL/LHDL ratio and TC/HDL-C ratios in all subgroups (*p* < 0.001). A similar correlation, albeit less significant, between the ratios of Apo B/Apo AI and sdLDL/LHDL of both dyslipidemic subgroups was observed (*p* < 0.05). Compared to NLs, an increase in plasma level of sdLDL was clearly prominent in both dyslipidemic subgroups. We performed receiver operating characteristic (ROC) analysis using plasma lipid profile and EMC content values to predict plasma level of sdLDL. The results of ROC analysis and AUCs for plasma TC, TG, HDL-C and EMC are shown in Table 5. TG and EMC levels had the highest AUC values in HCs. AUC values obtained in MTD group were not sufficient for statistical significant. As seen in Fig. 2, for prediction of plasma sdLDL in HCs, a value of 391 μg/mg prt. for EMC content showed optimum sensitivity (62 %) and specificity (64 %).

**Table 4:** Spearman rank correlations between ratios of lipids, lipoproteins, ghost C/P, and apoproteins

	TC/HDL-C (*r*, *p*)	TG/HDL-C (*r*, *p*)	Apo B/Apo AI (*r*, *p*)	sdLDL-C/LHDL-C (*r*, *p*)	Mean LDL Size (*r*, *p*)	EMC (*r*, *p*)
TC/HDL-C	–	0.664^a^***	0.499^a^***	0.261^a^***	−0.321^b^*	NS
		0.746^b^***	0.598^b^***	0.494^b^***	−0.392^c^**	
		0.483^c^***	0.534^c^***	0.505^c^***		
TG/HDL-C	–	–	0.579^b^***	0.312^a^*	−0.277^a^*	NS
				0.568^b^***	−0.484^b^***	
Apo B/Apo AI	–	–	–	0.501^b^*	−0.427^b^*	0.440^c^*
				0.389^c^*		
sdLDL-C/LHDL-C	–	–	–	–	−0.861^a^***	−0.386^b*^
					−0.951^b^***	
					−0.802^c^***	
Mean LDL size	–	–	–	–	–	0.257^a^*
						0.415^b^***

^a^Normolipidemic group, ^b^ hypercholesterolemic group, and ^c^ mixed-type dyslipidemic group

*p < 0.0 5, **p < 0.01, ***p < 0.001

**Table 5:** Area under the curve (AUC) for ROCs to predict high sdLDL-C status in dyslipidemic subgroups

Variable	Hypercholesterolemic group		Mixed-type dyslipidemic group	
	Area (95 % CI)	*p*	Area (95 % CI)	*p*
TG	0.757 (0.602–0.912)	0.005	0.591 (0.392–0.790)	0.368
TC	0.543 (0.361–0.726)	0.636	0.634 (0.447–0.821)	0.184
HDL-C	0.615 (0.442–0.787)	0.212	0.670 (0.495–0.844)	0.093
EMC level	0.768 (0.618–0.918)	0.004	0.396 (0.191–0.601)	0.302

## Discussion

In this study, the tendencies of individuals with lipidemia to develop atherogenic dyslipidemia were evaluated by analyzing lipid indexes, lipoprotein subfractions, and membrane lipid composition of erythrocytes. To our knowledge, this is the first study to report on a possible correlation between EMC content and subclasses of lipoproteins, i.e., LDL subfractions. A meticulous method for characterization of dyslipidemic profile is essential in diagnosis, prevention and treatment of cardiovascular diseases. More than 50 % the subjects with CAD have relatively normal blood LDL-C and HDL-C values hence several strategies have been developed for early diagnosis of lipid related risk factors in cardiovascular diseases (Mohan et al. 2005). Thus, dyslipidemic groups of our study were formed from individuals with mild lipid disorder.

Several studies have showed that there are higher level of sdLDL particles in the patients with lipid abnormality including metabolic syndrome, diabetes and CAD, conditions that are accompanied by dyslipidemia (Arai et al. 2013; Geiss et al. 2001; Kockx and Herman 2000; Kwon et al. 2006; Maeda et al. 2012; Musunuru 2010; Shoji et al. 2009; Superko 2009; Tziakas et al. 2008). In our study, sdLDL fraction was clearly increased in MTDs whereas LbLDL particle level was slightly decreased, although statistically insignificant in both dyslipidemic subgroups. These results are consistent with an earlier cross-sectional study showing higher sdLDL and Apo B levels in MTDs (Muth et al. 2010). Moreover, our result in HCs is also in agreement with reports showing LDL subfraction distribution in HCs being similar to those from NLs (Geiss et al. 2001). In present study, higher levels of TC, LDL-C, Apo B and lower level of HDL-C were found in subjects with elevated sdLDL particles (called pattern B), overlapped with atherogenic lipoprotein phenotype (data not shown).

It has been suggested that cardiovascular risk may be more closely related to atherogenic lipoprotein particle number and size rather than sdLDL-C level (Arai et al. 2013; Musunuru 2010; Shoji et al. 2009). Apo B values are directly correlated with LDL particle numbers and non-HDL fractions. Apo B/AI ratio, therefore, is more valuable than the standard LDL-C/HDL-C ratio in assessing risk factors for cardiovascular diseases and prediction of likelihood of cardiovascular complications (Arai et al. 2013; Kwon et al. 2006; Superko 2009). In this study, the highest Apo B/AI ratio was observed in MTDs, and this result was in agreement with findings from an earlier study (Superko 2009).

Analysis of HDL metabolism has been useful in determining the incidence and prevalence of atherosclerotic risks (Gou et al. 2005). Several studies showed that the antiatherogenic action of HDL involves a crucial player in reverse cholesterol transport (RCT) system and/or by an unidentified function involved in anti-inflammatory, antithrombotic, antioxidant and/or immunosuppressive systems. HDL subfractions exhibit a heterogeneous distribution based on density, size, and lipid and apoprotein compositions (Jia et al. 2007; Kockx et al. 1998). In our study, SHDL fraction value was higher in HCs whereas there was a decrease in LHDL fraction in both dyslipidemic subgroups, albeit statistically insignificant. SHDL fraction value in MTDs is similar to those observed in NLs. Given that mixed-type dyslipidemia is a strong risk factor for CVD events, these values contradicts the results of an earlier report showing higher level SHDL particles in MTDs compared to NLs (Jia et al. 2007). This contradiction could be explained by lower TG level of our group (239 vs. 374 mg/dL). It has been reported that plasma TG levels were positively correlated with SHDL level and negatively correlated with LHDL level in both dyslipidemic groups (Jia et al. 2007). It is also reported that CETP and HL activities induced by TG causes formation of the smaller HDL particles (Gou et al. 2005; Jia et al. 2007), which might explain why the lowest LHDL particles are found in MTDs. In sum, plasma TG seems to play a crucial role on HDL heterogeneity. Despite a strong correlation between CHD risk and HDL subfractions (Gou et al. 2005), several mechanistic studies have reported that LHDL fraction can be anti-inflammatory and thereby decrease CVD risk while SHDL particles may be proinflammatory and increase risk for recurrent CVD (Muth et al. 2010). Therefore, further studies are needed to elucidate molecular mechanisms underlying particular roles played by each HDL subfractions in atherogenic events.

Cholesterol content of erythrocyte membrane is 1.5–2 times higher than other cell types. A substantial amount of cholesterol in the necrotic lipid core of atheromatous plaques originates from erythrocyte membranes and from apoptotic macrophages whose apoptosis and resulting release of toxic lipid content is a flaming factor for inflammation and rupture of plaques (Kockx et al. 1998; Kockx and Herman 2000). Therefore, EMC content might be used as a marker for plaque stability in CAD (Tziakas et al. 2010; Zhang et al. 2011). Although statistically insignificant, our study indicated a linear increase in EMC content with the severity of lipidemia. EMC value of MTDs in comparison with other groups was the highest as other proatherogenic variables. There have been a number of case-control studies showing a significant increase in EMC level in dyslipidemic subjects with higher TC values (Koter et al. 2004; Uydu et al. 2012). Considering that EMC content is believed to be directly correlated with plasma cholesterol level, lower TC levels we report here is consistent with the observed EMC results in our study. Although the mechanism underlying the correlation between EMC and plasma lipoprotein levels remains unclear, it has been suggested that the exchange rate of cholesterol and phospholipids between erythrocyte and lipoproteins is regulated by several factors involving the number and distance of transfer sites among these particles as well as differences in C/P ratios of donor and acceptor particles (Rothblat and Phillips 2010; Tziakas et al. 2008, 2012). In particular, sphingomyelin content and lecithin and cholesterol acyltransferase activity of erythrocyte membrane regulate the rate of transfer of free cholesterol between cell membrane and plasma lipoproteins (Tziakas et al. 2010). Despite numerous reports on the relationship of erythrocyte to plasma lipid profile, the studies showing a possible interaction on membrane lipid composition and lipoprotein subfractions has been limited. Of these, it has been suggested on the basis of in vitro study that SHDL and LHDL subfractions function as EMC acceptor in subjects with normolipidemia and hypo-alpha-lipoproteinemia, respectively (Kozhevnikova et al. 1989). We observed no correlation between EMC content and HDL subfractions in all groups studied. However, a strong negative correlation between EMC and sdLDL was observed in HCs (*r* = −0.468, *p* = 0.002).

Clinical application of lipoprotein subfractionation is disadvantageous because it is costly and labor intensive (Kwon et al. 2006). Therefore, we calculated AUC values by ROC analysis using traditional plasma lipid profile analysis and EMC values for predicting plasma sdLDL level. Moreover, AUG values of TG and EMC in HCs may be advocated as a moderate surrogate marker for sdLDL.

We studied 148 subjects with normolipidemia and dyslipidemia. We assessed standard plasma lipid parameters, advanced lipid analysis and erythrocyte cholesterol content. Compared to normolipidemic subjects, a shift from larger toward smaller LDL and HDL particle diameters of both dyslipidemic subjects was observed. The relatively small number of subjects and the cross-sectional design of our study did not allow a comprehensive analysis on the metabolic pathways involved in the phenotypic expression of the dyslipidemic patterns.
